# Physical Functional Capacity and C-Reactive Protein in Schizophrenia

**DOI:** 10.3389/fpsyt.2016.00131

**Published:** 2016-08-05

**Authors:** Michele Fonseca Vieira Szortyka, Viviane Batista Cristiano, Keila Maria Ceresér, Lenise Petter Francesconi, Maria Inês Lobato, Clarissa Gama, Paulo Belmonte-de-Abreu

**Affiliations:** ^1^Post-Graduate Program in Psychiatry, Universidade Federal do Rio Grande do Sul (UFRGS), Porto Alegre, Rio Grande do Sul, Brazil; ^2^Department of Psychiatry, Hospital de Clínicas de Porto Alegre (HCPA), Universidade Federal do Rio Grande do Sul (UFRGS), Porto Alegre, Rio Grande do Sul, Brazil; ^3^Schizophrenia Program, Hospital de Clínicas de Porto Alegre, Porto Alegre, Rio Grande do Sul, Brazil

**Keywords:** schizophrenia, functional capacity, C-reactive protein

## Abstract

**Introduction:**

Schizophrenia is a severe, debilitating mental disorder that affects both the physical health and the functional capacity of patients, causing great impairment throughout the life course. Although physical and cognitive impairments may represent different expressions of a single systemic inflammatory process, little is known about the relationship between motor function and schizophrenia.

**Objective:**

To evaluate physical functional capacity in patients with schizophrenia and ascertain whether it correlates with markers of inflammation, disease severity, and pharmacotherapy.

**Methods:**

Cross-sectional study using a convenience sampling strategy. Forty patients with stable schizophrenia, undergoing treatment, were recruited from the Outpatient Program of Hospital de Clínicas de Porto Alegre, University Hospital linked to Public Health System. Physical functional capacity was assessed by the 6-min walk test (6MWT), and inflammatory markers were measured by C-reactive protein (CRP) and Von Willebrand factor.

**Results:**

Mean functional capacity and clinical variables differed among patients and Brazilian population regarding heart rate (*p* = 0.004), diastolic (*p* = 0.001) and systolic (*p* < 0.001) blood pressure, respiratory rate (*p* < 0.001), CRP (*p* = 0.015), Borg Scale of Perceived Exertion scores (BSPE) (*p* < 0.001), and 6MWT both in men (*p* < 0.001) and women (*p* = 0.024). Additionally, 6MWT and dyspnea in BSPE were positively associated with CRP (*r* = −0.369, *p* = 0.019) and *(r* = −0.376, *p* = 0.017) and (*r* = 0.354, *p* = 0.025 and *r* = 0.535, *p* < 0.001, respectively).

**Conclusion:**

The present study detected significant association between measures of functional impairment and markers of inflammation, especially elevated CRP in a group of stable outpatients with DSM-IV and ICD10 diagnosis of schizophrenia. Possible explanations for the associations could be linked to continued use of antipsychotics, although underlying neuroinflammatory mechanisms directly related to illness (schizophrenia) could not be ruled out. The findings of this study expand evidences of neuroinflammation to systemic inflammation in schizophrenia linking it to alterations of physical functional capacity and point to the need of additional studies exploring general inflammation and novel therapeutic interventions.

## Introduction

Despite increased morbidity and mortality in schizophrenia, patients still have limited access to health care and less opportunity to receive adequate prevention and treatment than non-schizophrenic population. Mean functional motor capacity is reduced in 20% compared to general population resulting in overall reduction of global functioning (1, 2).

Persons with schizophrenia have increased risk of obesity, probably due to sedentary lifestyle, inappropriate dietary choices, and adverse effects of psychiatric drugs (3, 4). Mortality is 2.4-fold higher than in the general population after excluding death from unnatural causes (5). Furthermore, studies show that patients with schizophrenia have limited access to general health care and fewer opportunities for prevention and treatment than would be expected in a non-psychiatric population (6).

The etiology of schizophrenia is still unclear, but several patients have reported abnormalities of immune response in patients with the condition, and have suggested that the neuroinflammatory response to immune reactions may play a crucial role in the pathogenesis of schizophrenia (7). There are strong hypotheses arguing for increased inflammatory markers due to microglia activation, causing abnormal neurogenesis, neural degradation, and white matter abnormalities, and thus playing a major role in the pathogenesis of schizophrenia (2, 8). An increase in proinflammatory cytokines, such as C-reactive protein (CRP), may also correlate with risk of metabolic disease and disease severity (9, 10), possibly including ability to perform physical tasks, integrate into social activities, perform activities of daily life (ADL), and increase overall quality of life (QOL) (11). Recently found that sedentary behavior is associated with increased CRP levels and marker of cardiovascular disease in people with psychosis (12). Patients with diagnosis of schizophrenia can perform inexpensive, easy, and rapid tests of functional capacity, like the 6-min walk test (6MWT), and provide relevant data about functional capacity (13, 14). The objective of the present study was to test if physical functional capacity in patients with schizophrenia correlates with common inflammatory markers, such as CRP and Von Willebrand factor (VWF). The main study hypothesis was that patients with schizophrenia would display association of raised levels of these markers with reduced functional capacity.

## Materials and Methods

The study displayed a cross-sectional design in a convenience sample of stable outpatients with simultaneous DSM-IVTR and ICD10 diagnosis of schizophrenia under public Health Care in an outpatient psychiatric clinic at a major teaching Hospital in Southern Brazil (Hospital de Clínicas de Porto Alegre – HCPA).

All participants had a confirmed diagnosis of schizophrenia, established over the course of at least four encounters involving information from both patient and their family members, with ages of 18–60 years, under stable psychopharmacological therapy tailored to their clinical condition. Patients were excluded if they had a history of alcohol or other drug abuse during the preceding month, a history of head trauma with posttraumatic amnesia, systemic or neurologic disease, current use of medication capable of inducing psychopathological manifestations, with present suicide risk, pregnant or lactating, with autoimmune conditions, taking steroidal anti-inflammatory drugs, or refusing to take part in the study. Participants completed the 6MWT, and underwent clinical assessment, and blood samples were collected at the HCPA Clinical Pathology Service as per routine laboratory practice. CRP was assayed by nephelometry (reference range: <5.0 mg/L = non-reactive; ≥5.0 mg/L = reactive), and vWF, by immunoturbidimetry (reference range: 50–160%).

Six-minute walk test was carried out in accordance with American Thoracic Society guidelines, along a hallway containing minimal external stimuli, with demarcated turnaround points, by two previously trained technicians. Participants were instructed to walk as briskly as possible, without running, during the 6-min test period. They were allowed to stop as necessary, but were notified that the timer would continue to run down. During the test, the technicians used standard words of encouragement. Blood pressure, heart rate (HR), respiratory rate, peripheral oxygen saturation, and dyspnea measured by Borg perceived exertion scale scores were measured both at the start and at the end of the test. Additionally, HR and blood oxygen saturation (BOS) were also measured at minute 3. Results were interpreted by means of the Enright and Sherrill algorithms for predicted 6MWD with upper and lower limits for sex, height, age, and weight.

Statistical analyses were carried out by SPSS 21.0 Software licensed to UFRGS. Variables were tested for normality by Kolmogorov–Smirnov test, and mean differences calculated by *t*-test for independent samples, Student’s *t-*test to compare sample means to general population, Chi-square test to analyze categorical variables, and Pearson or Spearman correlation coefficients as appropriate to test for associations among variables. The significance level was set at 0.05.

### Ethics Statement

The study was approved by the HCPA Research Ethics Committee with registry number 110083 in November 2011. Patients who agreed to take part in the study provided written informed consent. Furthermore, all patients were required to bring a chaperone or legal guardian signing the consent form and observe all data collection procedures.

## Results

**Figure 1:**
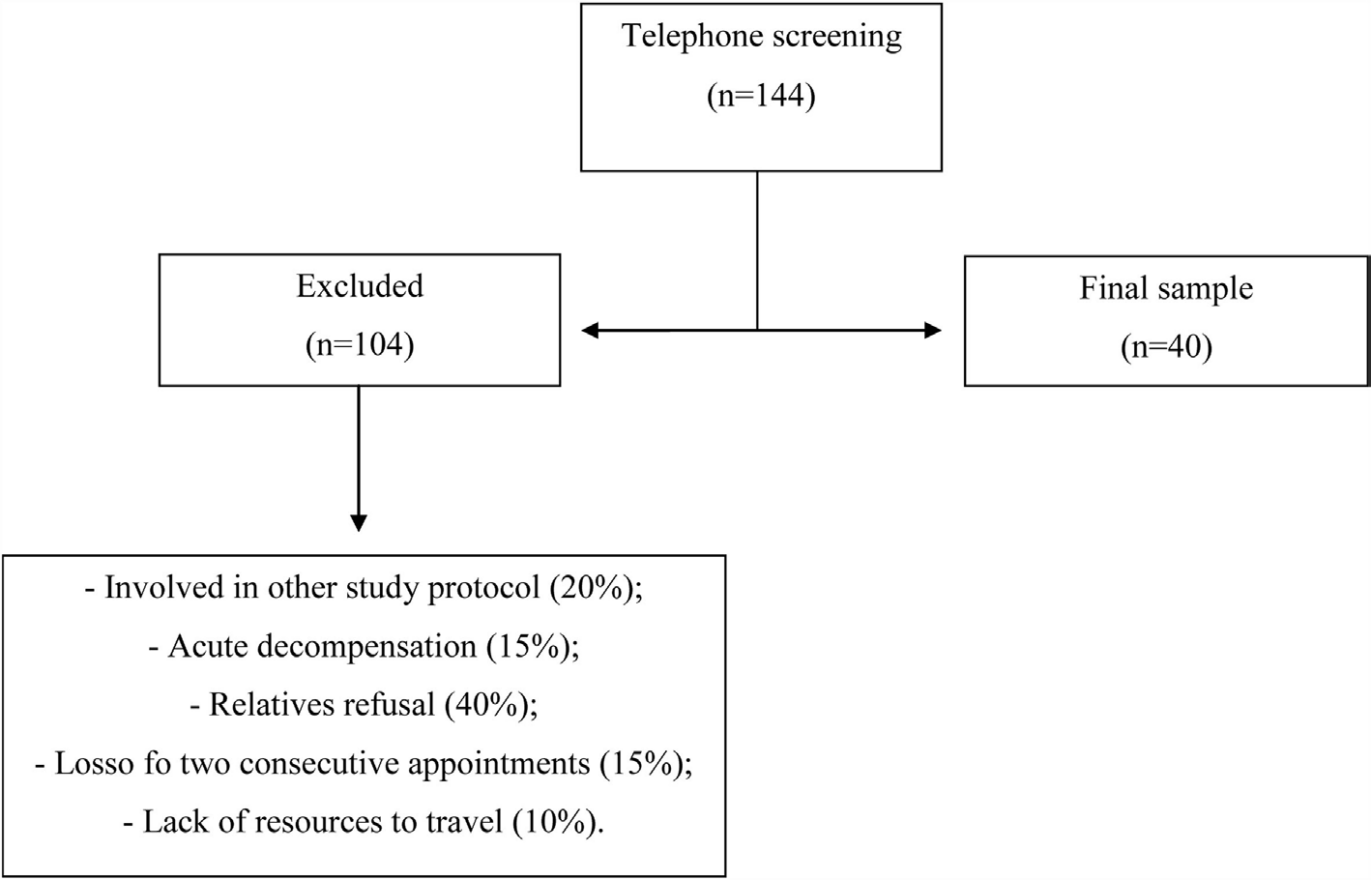
**Recruitment flowchart**. Patients with schizophrenia under regular care at the outpatient Program of Hospital de Clínicas de Porto Alegre (HCPA) recruited from January 2012 to December 2013.

From 415 patients in the HCPA Schizophrenia Program registry, 213 were not under present care. The 202 patients under regular care received a telephone-based screening interview, which excluded 162 patients due to study requirements, with a final sample of 40, 18% woman and 82% men (Figure 1). Socio-demographic characteristics were normally distributed and homogeneous across the two disease duration subgroups defined by data distribution (≤7 years and >7 years), as shown in Table 1. The only significant difference was found in marital status: patients in the >7-year disease duration group were more likely to be single or separated (Table 1).

**Table 1:** **Socio-demographic and clinical characteristics of outpatients with stable schizophrenia (*n* = 40)**.

Variable	Illness ≤7 years	Illness >7 years	Test	*p*
Age	32.83 ± 7.48	37.93 ± 8.64	Independent *t*	0.084
BMI	26.59 ± 5.26	27.51 ± 3.85	Independent *t*	0.539
Smoking	9/12	18/28	Chi-square	0.716
≤8 years Educ	4/12	14/28	Chi-square	0.268
Clozapine/other	8/12	21/28	Chi-square	0.704
Single/separated	9/12	28/28	Chi-square	0.022

*BMI, body mass index*.

*Disease duration (25th percentile = 7 years). Age and BMI normally distributed*.

Mean respiratory rate, BOS, blood pressure, and 6MWD in male and female patients were below measures of general population. HR and CRP and vWF levels were higher in clinical sample compared to general population (Table 2).

**Table 2:** **Comparison of mean clinical and functional variables in patients with stable schizophrenia (*n* = 40) and population averages**.

Variable (unit)	Cases (mean)	GP (mean)	Average difference (%)	*p*
HR (bpm)	92.00	80.00	15.00	0.004
Diastolic BP (mmHg)	74.50	85.00	12.35	0.001
Systolic BP (mmHg)	114.25	130.00	12.12	<0.001
BOS (%)	95.13	97.50	2.43	0.338
RR (bpm)	12.88	17.00	24.23	<0.001
CRP (mg/L)	6.18	4.00	54.50	0.015
vWF (%)	107.10	105.00	2.00	0.712
BDS scale 0–10	2.21	1.00	121.00	0.005
6MWT, males (m)	3.85	5.76	33.16	<0.001
6MWT, females (m)	4.04	4.94	19.22	0.024

*6MWT, 6-min walk test*.

*Population means: HR, heart rate (15); BP, blood pressure – diastolic and systolic (15); BOS, blood oxygen saturation (16); RR, respiratory rate (17); BDS, Borg dyspnea scale, and 6MWT, 6 min walking test (18)*.

*Population means for C-reactive protein (CRP) and von Willebrand factor (vWF) obtained from the HCPA laboratory (unpublished data)*.

Patients were stratified by disease duration according to 25th percentile, and medications in two categories (clozapine or other neuroleptics).

**Figure 2:**
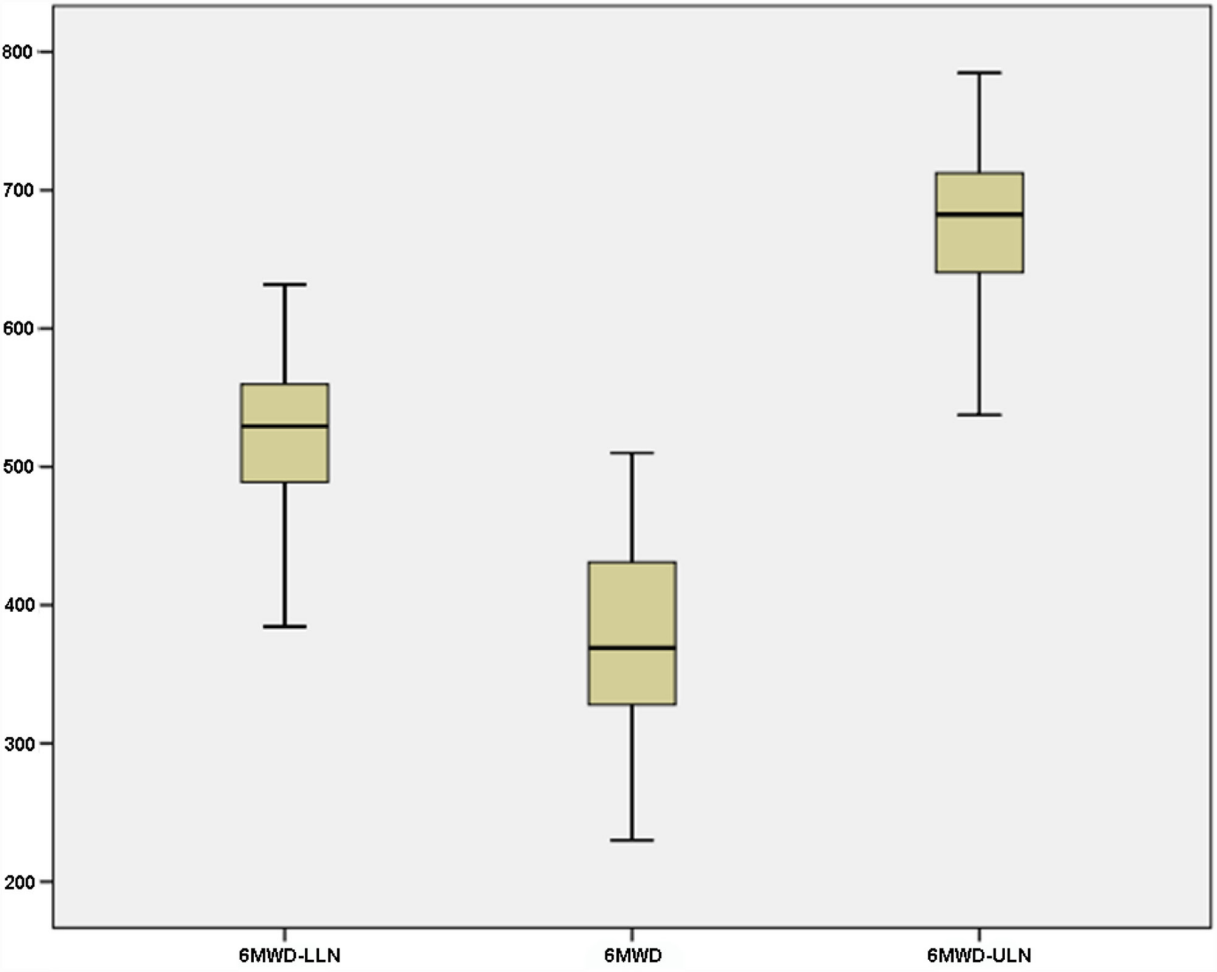
**Six-minute walking test (actual and predicted distance) in 40 patients with stable schizophrenia: 6MWD-LLN, predicted 6-min walk distance (lower limit of normal)**. 6MWD, actual 6-min walk distance. 6MWD-ULN, predicted 6-min walk distance (upper limit of normal). Predicted values were computed using the Enright algorithm (19).

Performance on 6MWD ranged from 0 to 610 m (Figure 2). Upper and lower limits of predicted 6MWD were 379.29–796.37 m and 240.29–643.37 m, respectively. Only 7.5% of patients (3 of 40) were reached the minimum predicted distance. Male mean 6MWD was 385 m, significantly lower than the population average of 576 m (*p* < 0.001), and Female performance was 404 m, also significantly above general population scores of 494 m (*p* = 0.024).

Correlation analysis of functional performance variables showed that impaired 6MWD (difference between actual and predicted distance) and dyspnea on the Borg scale were correlated to CRP (*r* = −0.369, *p* = 0.019 and *r* = −0.376, *p* = 0.017 and *r* = 0.354, *p* = 0.025 and *r* = 0.535, *p* < 0.001, respectively). These associations were stronger after controlling for smoking, age, and sex.

## Discussion

Although previous studies have identified reduced physical functional capacity in patients with schizophrenia by the 6MWT and other publications demonstrated the role of inflammatory markers in schizophrenia, this was the first study to detect association of reduced functional capacity (measured by HR, blood pressure, BOS, respiratory rate, and 6MWD) with increased inflammatory marker (CRP). By contrast, vWF, a reliable marker of endothelial dysfunction, were not significantly different from population levels, in contrast to Hope (17) of increased vWF in patients with schizophrenia compared to healthy controls (*p* < 0.05), increased vWF associated with small increase of CRP, and CRP and vWF positively associated with disease severity.

Body mass index and BP were not associated with physical functional capacity, suggesting that all patients were under adequate treatment, stable and well informed about illness and potential metabolic consequences. It is also of note that the usual treatment provided by the Outpatient Program includes dietary guidance.

In the sample of patients with schizophrenia included in this study, males and females had 30 and 20% reductions in functional capacity, respectively, corroborating Vancampfort (20) data of 20% reduction of 6MWD compared to healthy controls (*p* < 0.001). Additionally, Martín-Sierra (21) noted that impaired functional performance was associated with increased weight, metabolic complications, poor muscle fitness, smoking, negative symptoms, depressive symptoms, antipsychotic therapy, and reduced QOL. In the present study, smoking and antipsychotic use were not associated with reduced functional performance.

Dyspnea (Borg scale) in the sample was 121% higher than the national average. Vancampfort (20) found that severely mentally ill patients exhibited reduced forced expiratory volume in 1 s (FEV1) and forced vital capacity (FVC) compared to healthy controls, also associated with metabolic complications (22).

Among the different inflammatory markers, CRP holds a prominent position in severe psychiatric conditions, and particularly in schizophrenia. In the present study, elevated CRP levels were associated with functional impairment. In a 2014 review, Singh analyzed the current evidence for the role of CRP in schizophrenia, identifying increased CRP in 14 of 16 studies. Vuksan-Cusa (23) also reported elevated CRP levels associated with the risk of new onset metabolic syndrome. In the present sample, CRP levels were not associated with BMI (no other factors of metabolic disease were assessed in the study).

Dickerson (24), using a high-sensitivity CRP test (unlike conventional CRP testing as used in the present study), found elevated levels of CRP in patients with schizophrenia compared to healthy controls (*p* < 0.001). Comparison of bipolar patients disorder with normal controls failed to show difference, even after adjustment with logistic regression models.

Blood pressure was below the population average in the present sample. These results can be explained by the findings of Mackin (25), who reviewed cardiac effects of antipsychotic agents and reported association of clozapine therapy with orthostatic hypotension, probably due to the antagonist effect of clozapine with cholinergic and α1-adrenergic receptors, and with abnormalities of cardiac repolarization (specifically, prolonged ventricular repolarization). The same study also showed that clozapine associated with bradicardia, supporting the decreased HR observed in the present sample.

Overall, 72% of the 144 who completed the telephone interview did not participate due to failure to attend to the medical appointment in two consecutive days (only family members attended), recent participation in other studies, or some physical disability.

Limitations of the present study include the small sample size, failure to include additional clinical scales (BPRS, PANSS, Hamilton or Beck depression scales, etc.), the use of conventional CRP rather than high-sensitivity CRP assays, the lack of simultaneous assessment of metabolic variables (such as glucose, cholesterol, and triglyceride levels) and the lack of a control group. Furthermore, there was no record of specific medications used other than neuroleptics, and additional inflammation markers other than CRP and vWF, including cytokines and chemokines. It is advisable to surmount these deficiencies in future studies, including additional Clinical scales, inflammatory markers, and metabolic parameters.

In summary, the present study revealed an association between impaired physical functional capacity and increased CRP inflammatory marker in patients with schizophrenia, a complex condition with both physical and mental consequences. If reinforced by additional studies, it could also point to expanded treatment guidelines and protocols, including multidisciplinary and multi-systemic approach of patients with diagnosis of schizophrenia. Involvement of other professionals, such as physical therapists, trained to assess and treat functional impairments could then contribute to improved understanding and management of mental and physical health of patients with diagnosis of schizophrenia.

## Author Contributions

MS: data collection, manuscript drafting, and decision to submit the article for publication. VC: data collection. KC: data analysis. LF: data interpretation. MIL: data analysis. CG: data analysis. PB-d-A: data interpretation and manuscript drafting.

## Conflict of Interest Statement

The authors declare that the research was conducted in the absence of any commercial or financial relationships that could be construed as a potential conflict of interest.

## Disclosure

Fund for Research and Event Promotion of Clinical Hospital of Porto Alegre (Fundação de Incentivo à Pesquisa e Eventos, FIPE/HCPA) and Coordination for the Improvement of Higher Education Personnel (Coordenação de Aperfeiçoamento de Pessoal de Nível Superior, CAPES). These institutions participated in the analysis of study data.
